# Prevalence of Obesity in Elementary School Children and its Association with Dental Caries

**DOI:** 10.3290/j.ohpd.a43366

**Published:** 2020-04-01

**Authors:** Rodrigo Serrano-Piña, Fernando Javier Aguilar-Ayala, Rogelio José Scougall-Vilchis, Martha Liliana Trujillo-Güiza, Hugo Mendieta-Zerón

**Affiliations:** a PhD Student, Faculty of Odontology, Autonomous University of the State of México (UAEMex), Toluca, Mexico; Faculty of Odontology, Autonomous University of Yucatan (UADY), Yucatan, Mexico. Study concept and design, performed tests and statistical evaluation, wrote the manuscript.; b Professor, Faculty of Odontology, Autonomous University of Yucatan (UADY), Yucatan, Mexico. Proofread the manuscript, study sponsorship.; c Professor, Autonomous University of the State of México, Toluca, Mexico. Study concept and design, contribution to discussion.; d Professor, Antonio Nariño University, Colombia. Consulted on the statistical evaluation, contribution to discussion.; e Professor, Faculty of Medicine, Autonomous University of the State of Mexico (UAEMex); Ciprés Grupo Médico S.C. (CGM) and ‘Mónica Pretelini Sáenz’ Maternal-Perinatal Hospital (HMPMPS), Toluca, Mexico. Study concept and design, wrote the manuscript, design, statistical evaluation, study sponsorship.

**Keywords:** body mass index, carious lesions, children, O’Leary index, total decay

## Abstract

**Purpose:**

To determine any associations between obesity and caries activity in the mixed dentition stage among primary school children in a low-income Mexican primary school.

**Materials and Methods:**

This cross-sectional study was performed in Mexican schoolchildren aged 8–12 years. The body mass index (BMI) was obtained, and children were classified as overweight/obese considering age and sex. The experience of caries in permanent and temporary dentition was established with the sum of decayed/missing/filled teeth (DMFT or deft index for permanent or temporal dentition); a caries index for all teeth was also considered (total decay [TD]). Mann–Whitney U-test was used to contrast the distribution between sexes of the quantitative variables and to contrast the distribution of each variable per category, the Kruskal–Wallis test was used. Spearman’s Rho test was used to establish the correlation between the quantitative variables. Multiple linear regression models were performed to find the relationship between the O’Leary index and the BMI. A Multilayer Perceptron was constructed as follows: (a) dependent variables: deft, DMFT, TD and O’Leary index; (b) factor: BMI; (c) covariable: age.

**Results:**

A total of 331 children were included in the study. Dental caries prevalence was 32.4% (95% CI 29.7–35.2), while the mean DMFT was 0.64 (± SD 1.00). Through the Spearmen test, a statistically significant negative correlation was found between BMI-for-age with the total experience of carious lesions (r = –0.127, p = 0.021) and with experience of carious lesions in the deciduous dentition (deft) (r = -0.195, p ≤0.001). But when using the linear and logistic regression models to analyse the relationship with the O’Leary index, BMI was not statistically significant. With the Multilayer Perceptron there appears to be less error in the prediction of deft than the other indexes.

**Conclusions:**

This study confirms the high prevalence of obesity in primary school children. It also shows the scarce association between carious lesions and obesity.

Despite improved preventive regimens and advances in early diagnostic techniques, dental caries remains a highly prevalent childhood disease.^3,4^ Possible variables associated with dental carious lesions have been extensively investigated, and some are well known in children, such as sex, skin colour, diet, toothbrushing habits, along with socioeconomic factors, such as family income and type of school.^8,25^ Because high-calorie diets are a major cause of obesity, it is speculated that these diets could also cause dental caries and the explanation is based on the fermentable carbohydrates/sugar content of the diet. On the other hand, discussions about the relationship between dental carious lesions and other less studied factors such as obesity or physical activity have only increased recently.^24^ Numerous studies have been performed to determine whether an association exists between being overweight or obese and carious lesions; however, the results of these studies are inconsistent.^9,15,22,32^

Overweight and obesity are serious public health problems with particular concerns in children.^11^ The prevalence of obesity has increased rapidly worldwide in all ages and socioeconomic groups, both in developed and developing countries.^28^ In Mexico, obesity is a problem across all ages. In preschoolers, the prevalence of obesity was reported to be 34.4%.^26^ Although studies investigating obesity among Mexican students of primary schools have been published previously, there are no published works targeting the possible association between obesity, type of dentition and carious lesions. The aim of this study was to determine if any associations exist between obesity and carious lesion activity in the mixed dentition stage among school children in a low-income Mexican primary school.

## Materials and Methods

This was a clinical, descriptive, cross-sectional and prospective study. The targeted population included all of the children in the 4th, 5th, and 6th grade at the elementary school ‘Venustiano Carranza’ in San Mateo Atenco, State of Mexico. Third-grade schoolchildren aged 8–12 years were also selected to ensure that all subjects were in a stage of mixed dentition. Approval was obtained from the school principal prior to the school visit. Moreover, during the first visit, consent forms with information about the study were distributed to the students. Students were encouraged to return the form the following morning. A total of 400 consent forms were distributed. At the next school visit, the children who had brought back a signed consent form were examined. Children were eligible to participate if they had returned the signed parental consent form. All measurements were made between 8 and 11 am.

### Anthropometric Measurements

Height, weight, and waist circumference (WC) measurements of all of the students were recorded by a single examiner supported by an assistant for the registration of the obtained data. Height was recorded using a commercial, non-elastic measuring tape. The children stood barefoot on a flat surface, with their weight distributed on both feet, the heels together, with shoulders straight, arms hanging freely and head looking straight forward. A point was marked on a large white piece of cardboard fixed to the wall that corresponded to the highest point of the head. The tape was used to measure the distance from the floor to that point. Height was rounded to the nearest 0.1 cm.

An electronic weight scale (Microlife AG 9435 model) was used to measure body weight with light clothing and without shoes, jackets or heavy accessories. Each child was instructed to stand in the centre of the scale with the feet slightly apart and not to move until the weight appeared on the counter. The readings were rounded to the nearest 100 g.

WC was measured using a non-elastic measuring tape (Seca 201, Hamburg, Germany), at the highest point of the iliac crest when the child was standing at minimal respiration. The measurement was obtained with each child standing with the arms raised in a horizontal position. WC was rounded to the nearest 0.1 cm. Body mass index (BMI) was calculated for each student by dividing the weight in kilograms by the square of the height in meters (kg/m^2^).

In this study, BMI was categorised using the percentile charts for age and sex. The principal cut-off points and their corresponding classifications were as follows: underweight (<5th percentile), normal (5th–84th percentiles), overweight (85th–94th percentiles), and obese (≥95th percentile).

The z~score = ((observed value – median value of the reference population)/ standard deviation value of reference population) was calculated based on the values for Mexican children.^2^ With regard to WC, a cut-off of ≥90th percentile for age-specific and gender-specific measurements was used to define obesity.^18^

### Dental Evaluation

The dental evaluation was performed in the morning by a single examiner. The physical space of work was conditioned and organised previously. The intraoral evaluation was performed with natural light and the examiner used a lightweight, portable examination kit during the procedure. Children were seated in front of the examiner on a school chair during the examination. First, the amount of biofilm present in the oral cavity was counted. For biofilm staining, GC Plaque ID Gel dental plaque developer gel (GC NIMI Chemical Industrial, Yokohama, Japan) was used according to the manufacturer’s instructions. This material was applied with a regular 2 mm disposable microapplicator (Original Microbrush, Microbrush International, WI, USA). The excess developer gel was removed with purified water rinses for 15 s. The O’Leary index was used to calculate the percentage of stained surfaces in all the tooth/teeth elements present in the oral cavity.^20^

After the staining of biofilm, a dental prophylaxis treatment was performed to remove the remaining stains, plaque and food on the tooth surface, using a sterile brush, prophylactic paste, and a Saeshin electric micromotor (Saeshin Precision, Korea); the mouth was then rinsed for 30 s with cold purified water.

Later, the number of carious lesions in permanent and temporary dentition was established with the sum of decayed/missing/filled teeth (DMFT) or the deft index for permanent or primary dentition, which was considered a caries index for all teeth (total decay [TD]). A round-ended dental probe, a sterile, flat-surfaced non-magnifying mouth mirror (Hu-friedy Mfg. Co, LLC. USA), gauze and cotton rolls were used. The criteria for the diagnosis of the state of each tooth elements and the coding of the World Health Organization (WHO) were used.^34^ After the dental examination, a confidential report was sent to the parents informing them about their child’s oral health status and treatment needs.

### Statistical Analysis

Continuous data were expressed as means ± standard deviation (SD). Frequencies and percentages were reported for the categorical characteristics, as well as obesity status and carious lesions status in the primary and permanent teeth. The Kolmogorov–Smirnov/Shapiro–Wilks normality tests were performed on all quantitative variables. Mann–Whitney U-tests were used to contrast the distribution between the sexes for the quantitative variables. The Kruskal–Wallis test was used to test the distribution of the quantitative variables between the categories for each variable (TD, DMFT, deft, O’Leary, BMI-for-age and z~score). The Spearman’s Rho test was used to establish the correlation between the quantitative variables. Multiple linear regression models were performed to find the relationship between the O’Leary index and the BMI. The models considered the BMI as a continuous and categorical variable and the categories by means of z~score. Additionally, the same relationship was studied and regression models were carried out for the categories of the O’Leary index. Poisson linear regression models were used to evaluate the relationship between carious lesions and BMI. The associations between obesity status, carious lesions and gender were analysed using the chi-squared test, as were the associations between obesity status and carious lesions in the primary, permanent and both primary and permanent teeth. Pearson correlation was used to evaluate correlations between BMI and the evaluated indices (TD, deft, DMFT and O’Leary). The Multilayer Perceptron, which is one of the artificial neural networks^30^ used increasingly with more diversity in the clinical field,^27^ is a powerful interpolation mathematical ‘trainer’ system capable of calculating non-linear functions of any complexity from known input and output values used as examples for learning, consists of multiple layers including an input layer, multiple hidden layers and an output layer. In this project it was constructed as follows: (a) dependent variables: deft, DMFT, TD and O’Leary index; (b) factor: BMI; (c) covariable: age. In all cases, the statistical significance level was set at p <0.05, using SPSS version 23.0 software (IBM, Armonk, NY, USA).

### Ethics

The ethics committee of the ‘Mónica Pretelini Sáenz’ Maternal-Perinatal Hospital (HMPMPS), Health Institute of the State of Mexico (ISEM), approved the protocol (registered at the informatics platform of the ISEM, code: 217B500402016054). All procedures were performed in accordance with the Declaration of Helsinki and the General Health Law in Mexico. Informed consents were obtained with the signature of the parent or guardian and the student prior to performing any procedures.

## Results

The study included 331 elementary school children, 171 boys and 160 girls. The mean age of the participants was 10.2 ± 0.9 years, with a range of 8.1–12.4 years. Table 1 shows the general characteristics of the population: anthropometric data, BMI-for-age, caries experience (TD, DMFT, deft), and oral hygiene.

**Table 1:** Distribution of age, weight, height, BMI, DMFT, deft, TD and O’Leary index among the evaluated schoolchildren

	Total (n = 331)		Boys (n = 171)		Girls (n = 160)		P
	Mean ± SD	Range	Mean ± SD	Range	Mean ± SD	Range	
Age (years)	10.2 ± 0.98	8.0–12.4	10.2 ± 0.99	8.1–12.4	10.2 ± 0.9	8.1–12.2	-
Weight (km)	37.5 ± 9.8	19.6–87.7	30.1 ± 10.4	21.8–87.7	36.9 ± 9.2	19.6–66.8	0.548
Height (cm)	140.2 ± 8.3	121–168.5	140.2 ± 8.4	123.5–168.5	140.1 ± 8.2	121–160.5	0.890
BMI-for-age (kg/m2)	18.8 ± 3.5	11.8–33.0	19.1 ± 3.6	13–33	18.6 ± 3.4	11.8–31.3	0.406
DMFT	1.5 ± 1.9	0–11	1.4 ± 1.9	0–11	1.7 ± 1.9	0–11	0.057
Deft	3.6 ± 2.9	0–12	3.6 ± 2.9	0–11	3.5 ± 3.0	0–12	0.505
TD	5.1 ± 3.3	0–14	5.0 ± 3.3	0–14	5.2 ± 3.3	0–14	0.586
O’Leary Index	26.3 ± 6.1	12.5–52.2	26.1 ± 5.4	14.5–40.6	26.5 ± 6.6	12.5–52.1	0.761

SD, standard deviation; BMI, body mass index; DMFT, decayed, missed or filled permanent teeth; deft, decayed, extracted or filled primary teeth; TD, total decay.

The total number of teeth in the full population was of 7974 (boys = 4089, girls = 3885), divided into 2381 primary teeth (boys = 1312, girls = 1069) and 5593 permanent teeth (boys = 2777, girls = 2816). Table 2 contrasts the variables between genders. In comparison, weight, caries experience (DMFT, TD) and oral hygiene were slightly higher in women; however, these differences between sexes were not statistically significant. For the age range, the study population presented with mixed dentition; for instance, the mean tooth elements present in the oral cavity was 24.09 with a range of 20–28 (23.9 in males and 24.3 in females). Our results demonstrate that 70.14% of the examined teeth belonged to the permanent dentition. A statistically significant difference by sex was found in the total number of teeth (P = 0.026), as well as in the number of permanent teeth (P = 0.024), with females presenting higher number of erupted permanent teeth.

**Table 2:** Comparison of the evaluated variables among boys (n = 171) and girls (n = 160)*

			95% CI	
Variable	Type of analysis	P	Low	High
Age (years)	Equal variances assumed	0.805	–0.187	0.241
	Equal variances not assumed	0.805	–0.187	0.241
TD	Equal variances assumed	0.667	1.871	0.558
	Equal variances not assumed	0.667	–0.872	0.558
deft	Equal variances assumed	0.688	–0.509	0.770
	Equal variances not assumed	0.689	–0.510	0.771
DMFT	Equal variances assumed	0.159	–0.702	0.115
	Equal variances not assumed	0.159	–0.702	0.116
O’Leary Index	Equal variances assumed	0.566	–1.699	0.931
	Equal variances not assumed	0.568	–1.708	0.940
Weight (kg)	Equal variances assumed	0.296	–1.000	3.275
	Equal variances not assumed	0.294	–0.991	3.266
Height (cm)	Equal variances assumed	0.901	–1.691	1.920
	Equal variances not assumed	0.901	–1.690	1.919
BMI (kg/m^2^)	Equal variances assumed	0.222	–0.289	1.230
	Equal variances not assumed	0.222	–0.288	1.238
Primary teeth	Equal variances assumed	0.051	–0.003	1.985
	Equal variances not assumed	0.051	–0.005	1.988
Permanent teeth	Equal variances assumed	0.024	–2.543	–0.178
	Equal variances not assumed	0.025	–2.548	–0.173
Tooth/teeth elements	Equal variances assumed	0.026	–0.693	–0.045
	Equal variances not assumed	0.026	–0.694	–0.044

BMI, body mass index; deft, decayed/extracted/filled teeth; DMFT, decayed/missing/filled teeth; TD, total decay. *: Using the student t test.

Table 3 shows the distribution of the caries experience and oral hygiene index by categories of BMI-for-age and z~score. With regards to BMI-for-age, 54% of the population was classified as normal weight, 25.07% overweight, 13.89% obese and morbidly obese, and 6.64% underweight. The total experience of carious lesions was greater in the low weight and normal weight groups (TD 5.27 and 5.26, respectively). The overweight group showed a greater experience of carious lesions in permanent dentition (DMFT 1.69), while in the primary dentition it was greater in the underweight group (deft 4.0). Regarding oral hygiene, our study showed that the presence of biofilm was higher in the obesity and low weight categories (O’Leary 27.97 and 27.15, respectively), while schoolchildren with morbid obesity had a lower O’Leary index (25.4). No statistically significant differences were found in the caries experience and oral hygiene among the BMI-for-age categories.

**Table 3:** Distribution of caries experience, oral hygiene between categories of BMI-for-age and z~score

Classification	N = 331	TD			DMFT			deft			O’Leary Index		
		Mean ± SD	Range	P	Mean ± SD	Range	P	Mean ± SD	Range	P	Mean ± SD	Range	P
BMI-for-age category													
Low weight	22	5.27 ± 3.89	0–14	0.734	1.22 ± 1.82	0–7	0.658	4.0 ± 3.38	0–11	0.363	27.15 ± 4.0	19.32–36.46	0.156
NormalWeight	180	5.26 ± 3.23	0–13		1.48 ± 1.77	0–11		3.77 ± 2.93	0–12		25.96 ± 6.12	12.50–45.83	
Overweight	83	4.81 ± 3.41	0–13		1.69 ± 2.25	0–11		3.12 ± 3.0	0–10		26.92 ± 5.81	14.13–43.48	
Obesity	17	4.58 ± 2.85	1–9		1.05 ± 1.56	0–5		3.52 ± 2.15	0–8		27.97 ± 8.25	17.71–52.17	
Morbid obesity	29	4.86 ± 3.22	1–14		1.44 ± 1.7	0–5		3.41 ± 2.94	0–10		25.4 ± 6.34	14.58–38.54	
z~score category													
Low weight	19	4.73 ± 3.64	0–13	0.140	0.89 ± 1.28	0–4	0.555	3.82 ± 3.18	0–10	0.070	26.92 ± 3.6	19.32–32.29	0.189
NormalWeight	119	5.58 ± 3.2	0–14		1.47 ± 1.63	0–7		4.11 ± 3.0	0–12		26.41 ± 6.22	14.58–45.83	
Overweight	101	4.86 ± 3.24	0–13		1.59 ± 2.06	0–11		3.27 ± 2.88	0–10		27.11 ± 6.60	14.58–52.17	
Obesity	92	4.7 ±3.38	1–14		1.55 ±2.1	0–11		3.18 ± 2.82	0–10		27.11 ± 6.60	14.58–52.17	

BMI, body mass index; DMFT, decayed, missed or filled permanent teeth; deft, decayed, extracted or filled primary teeth; SD, standard deviation; TD, total decay

The distribution of the study population in the z~score categories was presented as follows: 35.95% were normal weight, 30.51% were overweight, and 27.79% were obese. The total caries experience was greater in the normal weight group (TD 5.58); the caries experience in permanent dentition was greater in the overweight group (DMFT 1.59), while in the primary dentition it was higher in the normal weight group (deft 4.11). The greater presence of biofilm was reported in the overweight and obesity groups with a mean of 27.11 but no statistically significant differences were found.

Through the Pearson test, a moderate negative correlation, which was statistically significant, was found between BMI-for-age with the total experience of carious lesions (r = –0.127, p = 0.021) and with experience of carious lesions in the deciduous dentition (deft) (r = –0.195, p ≤0.001) (Table 4).

**Table 4:** Pearson correlations of BMI with DMFT, deft, total decay and O’Leary index among all the evaluated schoolchildren

Variables	Pearson	P
DMFT and O’Leary index	0.157	0.004
BMI and DMFT	0.084	0.129
BMI and deft	–0.195	≤0.001
BMI and total decay	–0.127	0.021
BMI and O’Leary index	0.027	0.619

BMI, body mass index; DMFT, decayed, missed or filled permanent teeth; deft, decayed, extracted or filled primary teeth.

Linear and logistic regression models were adjusted to analyse the relationship between the O’Leary index and the other variables with a focus on the BMI. When an adjustment was made for sex and age, these variables were not statistically significant. Table 5 shows the results of the models adjusted for the O’Leary index, in all cases the independent variable ‘% permanent teeth’ was included. In models 1 to 3 the O’Leary index was processed as a continuous variable. While in model 1, BMI-for-age was considered as a continuous variable; in model 2 it was a categorical one. At last, in model 3 the categorical variable was the z~score category. In these three models the variable ‘% of permanent teeth’ was statistically significant (p ≤0.01).

**Table 5:** Regression models for O’Leary index

O’Leary index						O’Leary category*					
Model	Independent	C	95% CI	P	F	Model	Independent	C	95% CI	P	F
1	% permanent teeth	0.06	(2.66–9.49)	≤0.01	≤0.01^a^	4	% permanent teeth	1.02	(1–1.03)	≤0.01	0.03^a^
	BMI-for-age (continuous)	0.04	(–0.23–0.15)	0.68			BMI-for-age (continuous)	0.98	(0.92–1.05)	0.60	
2	% permanent teeth	0.06	(2.42–9.09)	≤0.00	≤0.01^a^	5	% permanent teeth	1.01	(1–1.03)	≤0.01	0.058
	BMI-for-age category						BMI-for-age category				
	Normal (reference)						Normal (reference)				
	Overweight	0.48	(–1.07–2.03)	0.54			Overweight	1.19	(0.71–2.01)	0.51	
	Obesity	0.21	(–1.71–2.14)	0.83			Obesity	1.22	(0.64–2.34)	0.55	
3	% permanent teeth	0.06	(2.76–9.39)	≤0.01	≤0.01^a^	6	% permanent teeth	1.02	(1–1.03)	≤0.01	≤0.01^a^
	Z~score category						Z~score category					
	Normal (reference)						Normal (reference)				
	Overweight	1.38	(–2.92–0.16)	0.08			Overweight	0.62	(0.36–1.06)	0.08	
	Obesity	0.29	(–1.29–1.88)	0.71			Obesity	1.03	(1.03–1.77)	0.91	

C, primary teeth/total teeth ratio coefficient; CI, confidence interval; BMI, body mass index. * Dichotomous variable. ^a^: chi-squared test.

When using the logistic regression models (4, 5 and 6), the O’Leary categorical variable was dichotomous, with models 4 and 6 being statistically significant overall. Interestingly, the ‘% of permanent teeth’ in the three cases was statistically significant in the same direction as the previous models; the BMI in its different forms was not statistically significant in any model.

Table 6 shows the models for the caries experience. Adjustments were tested for the variables; however, the majority of variables were not statistically significant. Only the percentage of permanent teeth showed statistical significance. Therefore, the final model considered three variables: percentage of permanent teeth, O’Leary index and BMI. The O’Leary index was statistically significant in all three models, with a relative risk (RR) = 1.02 (95% CI: 1.01–1.02, p ≤ 0.01). The percentage of permanent teeth was statistically significant in all three models (p ≤0.01). No statistically significant relationship with BMI was found in any of the models.

**Table 6:** Poisson regression models for caries experience

	Independent	RR	CI _95%_	P	F*
Model 1	% permanent teeth	0.99	(0.99–0.99)	≤0.01	≤0.01
	O’Leary	1.02	(1.01–1.02)	≤0.01	
	BMI-for-age (continuous)	0.99	(0.98–1.01)	0.23	
Model 2	% permanent teeth	0.99	(0.99–0.99)	≤0.01	≤0.01
	O’Leary	1.02	(1.01–1.02)	≤0.01	
	BMI-for-age		(0.0)		
	Normal (reference)		(0.0)		
	Overweight	0.97	(0.86–1.09)	0.58	
	Obesity	0.90	(0.78–1.05)	0.17	
Model 3	% permanent teeth	0.99	(0.99–0.99)	≤0.01	≤0.01
	O’Leary	1.02	(1.01–1.02)	≤0.01	
	Z~score category		(0.0)		
	Normal (reference)		(0.0)		
	Overweight	0.96	(0.86–1.08)	0.49	
	Obesity	0.91	(0.81–1.02)	0.11	

BMI, body mass index; CI, confidence interval. *chi-squared test.

With the Multilayer Perceptron to investigate relationships between carious lesions and oral hygiene the average overall relative errors were fairly constant across the training (0.957) and testing (0.911) samples, which give us some confidence that the error in future cases, scored by the network will be similar to this model. It can be seen also that there appears to be less error in the prediction of deft than the other indexes in the primary school children as the relative error in both, the training (0.883) and testing model (0.795) had the lowest values (Table 7).

**Table 7:** Multilayer Perceptron analysis to investigate relationship between carious lesions and oral hygiene

Model summary			
Training	Sum of squares errors		432.738
	Average overall relative error		0.957
	Relative error for scale dependents	DMFT	1.002
		deft	0.883
		Total decay	0.948
		O’Leary	0.997
Tests	Sum of squares errors		62.110
	Average overall relative error		0.911
	Relative error for scale dependents	DMFT	0.875
		deft	0.795
		Total decay	1.073
		O’Leary	0.980

DMFT, decayed, missed or filled permanent teeth; deft, decayed, extracted or filled primary teeth.

## Discussion

Several reports have shown that the prevalence of being overweight or obese among children is increasing in developed and developing countries, and this issue is becoming a public health concern.^7,13^ It is plausible that a high BMI can be harmful to the dental health status of a child.^21^ On this subject, sugar-sweetened, carbonated beverages have been found to be associated with a higher BMI and poor dietary choices involving, for example, frequent desserts, savoury snacks and total sugar consumption, as well as with lower milk consumption.^5^ Our study showed no statistically significant difference in the prevalence of obesity between boys and girls based on BMI and agrees with the findings of the 2015 National Health and Nutrition Examination Survey.^19^

Obesity and carious lesions are concomitant conditions in many populations, largely because of common risk factors, including consumption of highly calorific and cariogenic substances. Regarding oral hygiene, the heterogeneous results of our population reinforces the fact that, although qualitative differences in the prevalence of dental caries among BMI and z~score groups were identified, in fact, no statistical differences have been found.

Our data add to the reports of higher the prevalence of obesity in low socioeconomic status groups.^17^ Even more, this finding is consistent with reports of significantly higher percentages of carious lesions in older children.^23^

The current data show an association between anthropometric variables, especially between BMI and carious lesions, but there has been conflicting evidence in the literature with regard to the nature and direction of this association. Similar to our results, it has been shown that dental carious lesions are inversely associated with all anthropometric outcomes in Saudi children, including height and weight, suggesting that untreated carious lesions are associated with poorer growth.^1^ Furthermore, a survey conducted in 2012 showed that a smaller proportion of obese children present with dental caries at their dental initial examinations compared with normal weight children.^31^

Some effects of dental carious lesions on weight homeostasis that should be considered with regard to reduced weight gain are pain while chewing,^1^ and alterations due to immune, endocrine or metabolic responses.^12^ The relationship in our sample population is not clear, as there was an inverse relationship between the BMI and the presence of carious lesions, possibly explained by the primary teeth change.

Willershausen et al found a significantly lower percentage of obese children to have carious lesions-free teeth than normal weight children.^32,33^ Further, an association between high BMI and incidence of permanent molar smooth surface carious lesions was documented in another study.^14^ Nevertheless, some investigators have found no correlation between childhood obesity and dental carious lesions.^16,29^

Innovating in the study approach of obesity and carious lesions using the Multilayer Perceptron analysis, which determines different strengths of influence between the variables characterised by weights, showed that BMI had a less error for prediction of deft than for DMFT, TD and O’Leary index.

Clearly, diet plays a key role in the development of both obesity and dental carious lesions. For example, healthy eating, with the inclusion of fruits and vegetables, promotes better overall health. As such, children who make good food choices, including vegetables, experience lower risk of carious lesions.^10^ At the time of writing, the specific analysis of the effect of each group of nutrients on the presence of carious lesions was beyond the scope of this study.

Fortunately, dental carious lesions are mostly preventable, and even reversible if detected in early stages and if effective intervention is available.^6^ It seems that public intervention against carious lesions in children has not been as effective as expected; this is in part due to the conditions and variations in the implementation of the oral health programme for schoolchildren in Mexico. For example, it is common for health promoters not to consider the indications for the application of topical fluoride in schools, such as previous training in children to retain fluoride in the mouth for 1 min, the necessity of the child to have control of the swallowing reflex and even the preparation of the fluoride solution that will be applied, as well as the correct amount of fluoride for each child. The incorrect combination of these situations will decrease the efficacy of topical fluoride application in mixed dentition.

Some limitations of this study are the lack of information about nutritional habits, socioeconomic status and the activity level of the children. Furthermore, no radiographs were used to confirm the presence of carious lesions.

## Conclusion

This study confirms the high prevalence of obesity in schoolchildren and its scarce association with the presence of carious lesions. In conclusion, the behaviour observed with the oral indices shows a better prediction of deft index. Opportunities for future research would be beneficial in the fields of neural networks and health processes as carious lesions prevalence is very diverse, including data preprocessing and representation, architecture selection and application. An improved input would help in discerning which molecular pathways predispose to carious lesions.

Future studies containing larger samples are needed to evaluate the effect of obesity on specific types of carious lesions (fissure versus smooth surface carious lesions and anterior versus posterior carious lesions). Furthermore, investigators are encouraged to further evaluate the effect of ‘junk food’ on carious lesions prevalence depending on the child’s BMI.
